# Synthesis of Bioactive Microcapsules Using a Microfluidic Device

**DOI:** 10.3390/s120810136

**Published:** 2012-07-26

**Authors:** Byeong Il Kim, Soon Woo Jeong, Kyoung G. Lee, Tae Jung Park, Jung Youn Park, Jae Jun Song, Seok Jae Lee, Chang-Soo Lee

**Affiliations:** 1 Center for Nanobio Integration & Convergence Engineering (NICE), National Nanofab Center, 291 Daehak-ro, Yuseong-gu, Daejeon 305-806, Korea; E-Mails: kbiset@nnfc.re.kr (B.I.K.); swjeong@nnfc.re.kr (S.W.J.); kglee@nnfc.re.kr (K.G.L.); 2 Department of Chemical Engineering, Chungnam National University, 220 Gung-Dong, Yuseong-gu, Daejeon 305-764, Korea; 3 Department of Chemistry, Chung-Ang University, 84 Heukseok-ro, Dongjak-gu, Seoul 156-756, Korea; E-Mail: tjpark@cau.ac.kr; 4 Biotechnology Research Division, National Fisheries Research & Development Institute (NFRDI), 408-1 Sirang-ri, Gijang, Busan 619-705, Korea; E-Mail: jypark@nfrdi.go.kr; 5 Microbe-based Fusion Technology Research Center, KRIBB, 1404 Sinjeong-dong, Jeongeup, Jeonbuk 580-185, Korea; E-Mail: jjsong@kribb.re.kr

**Keywords:** microcapsulation, NIPAM, hydrogel, microfluidic device, spore

## Abstract

Bioactive microcapsules containing *Bacillus thuringiensis* (*BT*) spores were generated by a combination of a hydro gel, microfluidic device and chemical polymerization method. As a proof-of-principle, we used *BT* spores displaying enhanced green fluorescent protein (EGFP) on the spore surface to spatially direct the EGFP-presenting spores within microcapsules. *BT* spore-encapsulated microdroplets of uniform size and shape are prepared through a flow-focusing method in a microfluidic device and converted into microcapsules through hydrogel polymerization. The size of microdroplets can be controlled by changing both the dispersion and continuous flow rate. Poly(*N*-isoproplyacrylamide) (PNIPAM), known as a hydrogel material, was employed as a biocompatible material for the encapsulation of *BT* spores and long-term storage and outstanding stability. Due to these unique properties of PNIPAM, the nutrients from Luria-Bertani complex medium diffused into the microcapsules and the microencapsulated spores germinated into vegetative cells under adequate environmental conditions. These results suggest that there is no limitation of transferring low-molecular-weight-substrates through the PNIPAM structures, and the viability of microencapsulated spores was confirmed by the culture of vegetative cells after the germinations. This microfluidic-based microencapsulation methodology provides a unique way of synthesizing bioactive microcapsules in a one-step process. This microfluidic-based strategy would be potentially suitable to produce microcapsules of various microbial spores for on-site biosensor analysis.

## 1. Introduction

Recently, the generation of spatially well-defined, three dimensional (3D) microstructures for whole-cell sensing system have attracted interest in the development of portable bacterial whole-cell biosensing systems, high-throughput cellular analysis as well as in fundamental studies of cell biology [1–3]. To achieve this goal, three major aspects should be considered: (a) selection of biocompatible materials to construct 3D microstructures; (b) fabrication methods to control the size and uniform shape of the 3D microstructures; (c) polymerization methods to produce hydrogels [4–6]. In the aspect of selection of biocompatible materials, different types of biocompatible materials have been used over the past few decades. Among the various types of biocompatible materials, hydrogels have been attractive to biochemical, biomedical and biomaterial researchers because of their non-toxic, robustness and inertness properties. Among the different kinds of hydrogel materials, including polyethylene glycol (PEG), alginate and poly(N,N-dimethylacrylamide) (PDMAA), the hydrogel based polymer poly(*N*-isoproplyacrylamide) (PNIPAM), has been one of the most promising materials for the manipulation of biomaterials [7–10]. In addition, unlike other hydrogels, PNIPAM produces a thin wall which provides a unique hollow inner structure. Because of this unique structural advantage, microorganisms could be encapsulated inside the resulting microcapsule and it could provide enough space for their growth [7,11].

The fabrication method used to generate 3D microstructures of uniform size and shape is another important factor. In order to achieve these goals, microdroplet-based microfluidic systems have been developed and widely used [12,13]. With the advanced technologies for the transport and manipulation of droplets, many possibilities exist nowadays to carry out synthesis and functionalize microdroplets for biomedical applications, including therapeutic delivery and biomedical imaging, biotransformation, diagnostics, and drug discovery [3,14–16]. For this reason, microdroplets have been widely employed as a container to encapsulate various types of biological substances (*i.e.*, cells, DNAs, and proteins) in discrete microdroplets [7,12,16].

Unlike conventional systems, the selection of materials for the production of microfluidic devices is important for the generation of microdroplets of uniform shape. Among several materials, polydimethylsiloxane (PDMS) has been widely used as a material of choice to produce microfluidic devices due to the low-cost fabrication of the microfluidic channels, high transparency, and biocompatibility. The polymerization method of a monomer mixture in microdroplets is also important to produce the hydrogels [7,9]. Previous studies show the great potential fabrication method using UV irradiation in many fabrication methods, especially in the fabrication of polymer particles. However, the polymer residues are normally stacked in the orifice or microchannels and they disrupt the flow inside of PDMS channels rendering difficult the stable production of particles of uniform size. In addition, the PDMS itself adsorbs UV light, preventing proper curing of polymers [17]. In order to overcome this issue, chemical polymerization has been developed and applied to fabricate hydrogels [8,18].

In this study, we have developed a new method to produce bioactive monodisperse PNIPAM-based microcapsules by using a combination of a microfluidic device and a chemical polymerization method. Monodisperse microdroplets were formed by two immiscible fluids in a flow-focusing microfluidic device. The polymerization process of continuously producing microdroplets was initiated by the addition of *N,N,N*′,*N*′-tetramethylethylenediamine (TEMED) which acts as a catalyst. In addition, the produced microcapsules were highly monodispersed and suitable for the mass production of microcapsules at room temperature with easy size control. To further demonstrate the ability of the synthesized PNIPAM-based microcapsules to act as bioactive containers, *BT* spores were employed, and encapsulated inside the microcapsules and subsequently cultivated inside the microcapsules for shuttling to the vegetative cells.

## 2. Experimental Section

### 2.1. Materials

*N*-isopropylacrylamide (NIPAM, 99%), *N,N*′-methylenebisacrylamide (MBA, 99%, crosslinker), potassium persulphate (KPS), TEMED (99.9%), isopropyl alcohol (IPA, 99.9%), urografin and phosphate-buffered saline (PBS) solution were purchased from Sigma-Aldrich (St. Louis, MO, USA) and used without further purification. PDMS (Sylgard 184, Dow Corning, Midland, MI, USA) and SU-8 photoresist (Microchem, Newton, MA, USA) were used for the fabrication of PDMS microfluidic device. Refined grape-seed oil (G-oil, Beksul, Seoul, Korea) and Abil EM90 (Degussa, Essen, Germany) were mixed and used as a continuous flow solution.

### 2.2. Preparation of Bacterial Spores

All bacterial strains and plasmids used in this study were reported previously [19]. We cultured *BT* subspecies *israelensis* 4Q7 harboring expression vector for displaying proteins in CDSM media [20] at 37 °C with 250 rpm for 48–60 h. Pellet was obtained from 100 mL of culture by centrifugation (10,000 × *g*, 10 min) and suspend the pellet in 1 mL of 20% (wt/vol) urografin. This suspension was gently layered over 10 mL of 50% (wt/vol) urografin in a 15 mL centrifuge tube, and then centrifuged for 4 °C, 10 min at 16,000 × *g*. The collected pellets containing only free spores were stored at −20 °C [20,21].

### 2.3. Fluorescence Analysis

The purified EGFP-displaying *BT* spores by the urografin gradient method [20] were washed and resuspended at ∼1.0 × 10^8^ CFU/mL in PBS. Fluorescence assay was performed using a multi-plate reader, SpectraMax M2 (Molecular Devices, Sunnyvale, CA, USA). Flow cytometry data was obtained using a FACSCalibur™ flow cytometer and the Cell Quest Pro™ software (BD Bioscience, San Jose, CA, USA). Spores displaying EGFP was analyzed for relative fluorescence intensity using an FL1 green fluorescence detector with a 530/30 nm bandpass filter. The mean value (M) indicates the mean fluorescence intensities obtained by FL1 detectors.

### 2.4. Imaging of EGFP-Displayed Spores

The purified EGFP-displaying spores were mounted on poly-L-lysine-coated glass slides (Cel & Associates, Pearland, TX, USA) and analyzed under an LSM 510 confocal laser scanning microscope (Carl Zeiss, Göttingen, Germany). Samples were excited at 488 nm with an argon laser, and the images were filtered with a longpass 505 nm filter. All final images were generated from 4–5 serial images made by automatic optical sectioning.

### 2.5. Fabrication of PDMS Microfluidic Devices

PDMS/PDMS bonded microfluidic channel designs were fabricated by soft lithography and PDMS molding technique. The silicon master was coated with SU-8 photoresist by spin-coating and transferred the design onto the wafer using the mask and UV light exposure. Microfluidic devices were obtained with PDMS using silicon master with SU-8 pattern. A mixture of PDMS prepolymer and curing agent (10:1 Sylgard184, Dow Corning) was stirred and degassed in a vacuum oven at 70 °C. After curing, the PDMS replica was peeled away from the silicon master then bonded with another PDMS using O_2_ plasma.

### 2.6. Droplet Polymerization and Spore Germination

The droplets were generated using the microfluidic device with a flow-focusing technique. The dispersive phase (DP) consisted of the mixture of potassium persulphate (initiator, 0.19 wt%), D-sorbitol (cross-linker, 0.6 wt%), PBS solution (56 wt%), NIPAM (24.8 wt%) and *BT spores* and LB broth (0.18 wt%). The continuous phase (CP) is the mixture solution of G-oil and Abil EM90 (2 wt%). The microdroplet generation in the microfluidic device was observed using an optical microscope with a charge-coupled-device camera (Elipse Ti-S, Nikon, Tokyo, Japan). Once the microdroplets were generated through flow-focusing, the microdroplets were collected and suspended in TEMED/G-oil mixture (7.7 vol%) for the polymerization. TEMED is acted as a catalyst for encouraging the polymerization and produce hydrogel microcapsules. In addition, the Abil-EM90 was used as a surfactant to prevent the coagulation between the generated microdroplets during the polymerization. The polymerized spore encapsulated microcapsules were washed with IPA and PBS solution several times and then dispersed in LB medium and stored for overnight. A confocal microscope (LSM510 META NLO, Carl Zeiss, Göttingen, Germany) was used to monitor fluorescence intensity changes of *BT* spores in the PNIPAM microcapsules.

## 3. Results and Discussion

**Figure 1.**
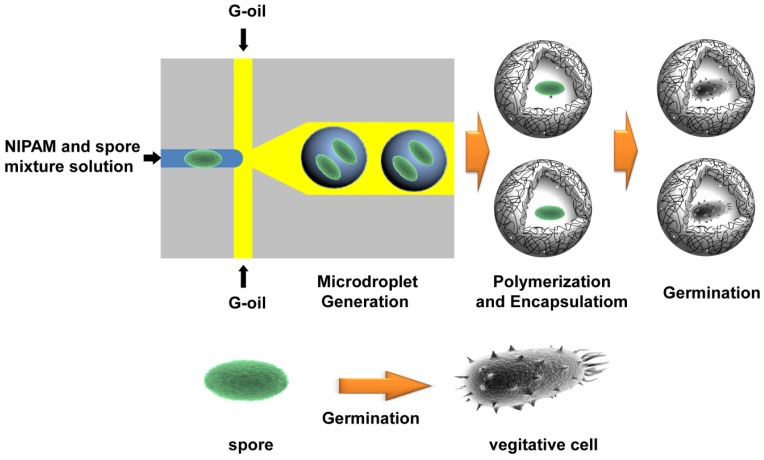
Schematic illustration of the *BT* spore-encapsulation process and the germination of *BT* spores in the microcapsules.

The major dimensions of microfluidic device were 50 μm of orifice and 100 μm of height for all microchannels, and the detailed dimensions of the microfluidic device and its picture are shown in Figure S1. For the production of microdroplet-based hydrogel beads, the mixture of NIPAM (20%, w/w), MBA (5%, w/w), initiator, and mixture solution of EGFP-displayed *BT* spores (1.0 × 10^5^ CFU/mL) were injected through the center inlet of PDMS-based microfluidic device as a DP. In order to generate microdroplets, a mixture of G-oil and Abil Em90 as a surfactant was employed as a CP through the other inlets. The overall fabrication processes and the dimension of microfluidic device are schematically illustrated in Figures 1 and S1. In this study, the G-oil and Abil Em90 were selected because it is inert, immiscible with PNIPAM monomer and prevents the potential merging of produced microdroplets. As DP passing through the orifice of the device, the DP flow is squeezed and sheared off by applied CP flow and the orifice to form monodisperse microdroplets.

**Figure 2.**
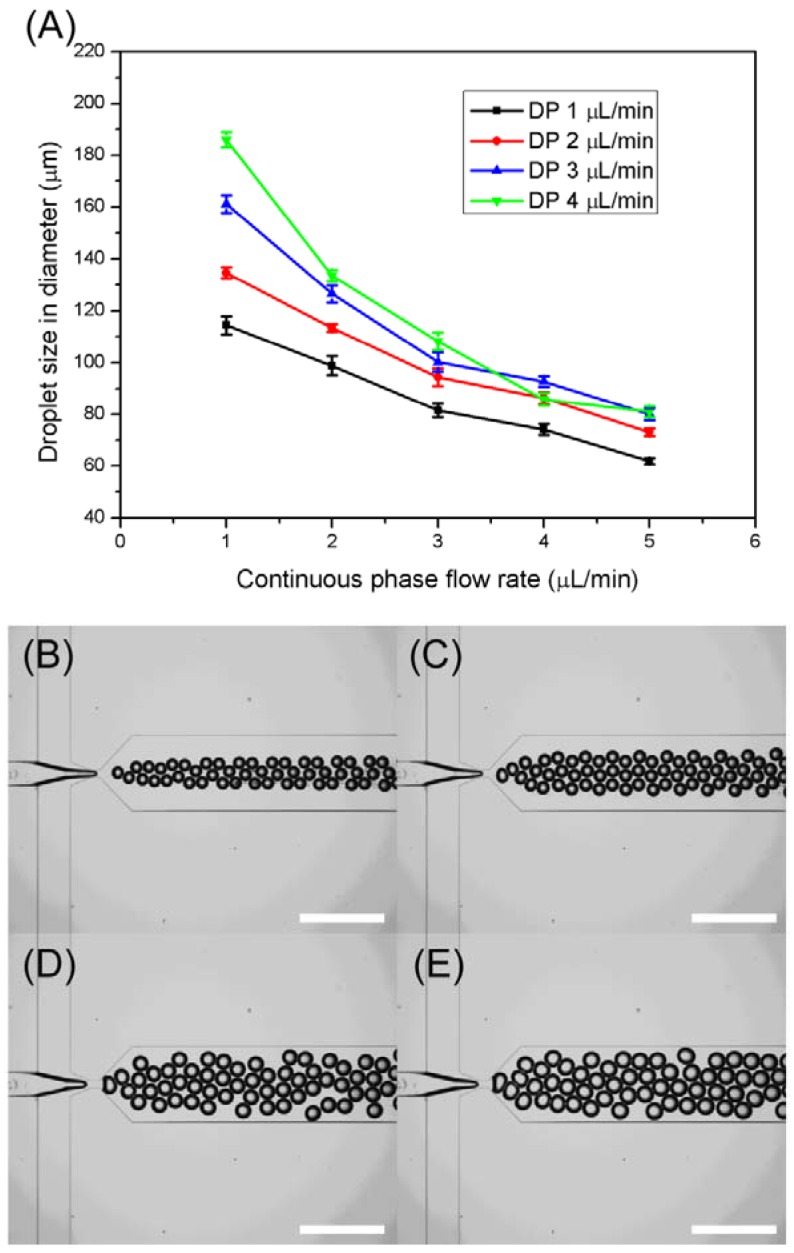
(**A**) Droplet size variation graph with both different flow rates of CP flow rate (1–5 μL/min) and DP (1–5 μL/min). Optical microscopic images of microdroplet generation at the fixed CP (2 μL/min) with four different flow rates of DP. (**B**) *Q*_DP1_ = 1 μL/min, (**C**) *Q*_DP2_ = 2 μL/min, (**D**) *Q*_DP3_ = 3 μL/min, (**E**) *Q*_DP4_ = 4 μL/min.

The production of different sizes of bioactive microcapsules is important to control the amount of loading of biomolecules, cells or biomaterials for further applications. For this reason, the microdroplet-based microfluidic device was fabricated for controlling the size of microcapsules. The size of microdroplets was simply controlled by changing of both CP and DP flow rate in the microfluidic device, and the results are demonstrated in both Figure 2 and Figure S1–S4. Figure 2(A) shows the relationships between the droplet size and CP and DP flow rates in the microfluidic device. Under the same CP flow rate, the high-flow rate of DP generates the relatively large size of microdroplets compared to the low-flow rate of DP. Moreover, as increasing the CP flow rate under the same DP flow rate, the size of produced microdroplets is decreased. These results indicate that high-flow rate of CP strengthens the shearing force and accelerate the detachment of the droplets from the DP flow at the orifice in the microfluidic device. Because of this mechanism, a broad size range of microdroplets from 186 μm to 61 μm was easily obtained using a microfluidic device by controlling both CP and DP.

The detailed results of microdroplet production using the microfluidic device under different flow rate conditions are shown in Figures S2–S5. The CP and DP flow rates were easily controlled by a syringe pump. Even though the generated microdroplets were close to each other, each droplet was separated with each other due to the presence of surfactant as shown in Figure 2(B–E). Unlike the UV-based polymerization method, no surface discoloration of PDMS, which is an indication of the PDMS damage by UV irradiation, were observed during the chemical polymerization. Moreover, there was no blockage of microchannels or orifices in the microfluidic device by polymer residues, which is a common occurrence during UV-based polymerization processes. These stable conditions lead to continuous production of *BT* spores-encapsulated microcapsules through the microfluidic devices.

Once the microdroplets were formed through the orifice, the obtained microdroplets were merged in the mixture of TEMED which is acted as a catalyst. As soon as the mixture of NIPAM monomer, initiator, crosslinker, and *BT* spores in the microdroplets exposed to the TEMED, NIPAM monomers start to polymerize through the chemical polymerization. During the polymerization, the potassium persulfate as an initiator induced the radicals, and NIPAM was crosslinked with MBA as a crosslinker and physically enclosed the *BT* spores in the PNIPAM microcapsules. In the most of the cases, the shape and size of hydrogels in microdroplets are changed during the polymerization process. For the investigation of morphological changes microdroplet during the polymerization process, we selected the DP flow rate as 1 μL/min and the CP flow rate as 5 μL/min, respectively.

**Figure 3.**
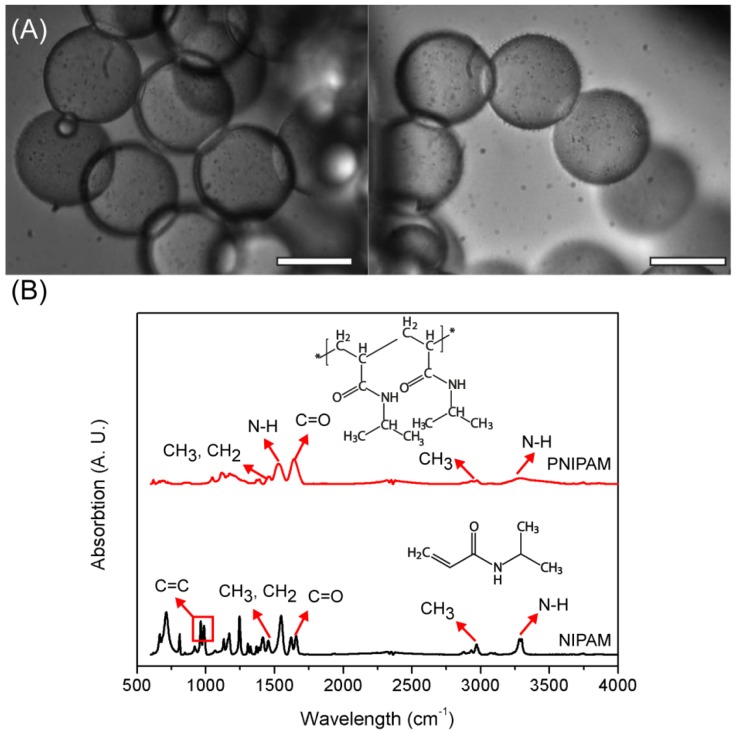
(**A**) Microscopic images of polymerized PNIPAM microbeads. Scale bars are 50 μm; (**B**) FT-IR spectra of NIPAM and PNIPAM.

As shown in Figure S1, the average produced droplet size was around 62 μm. After the produced microdroplets were polymerized, the monomers inside the droplets were polymerized and microcapsules were fabricated. During the polymerization process, the microcapsules maintained their uniform spherical shapes even after the polymerization, as shown in Figure 3, and the average produced PNIPAM microcapsules were 60.29 ± 2.19 μm in diameter, which is similar to the average size of microdroplets. There are slight changes of the diameter, and no shape changes were observed, as shown in Figure 3. This result demonstrates the successful fabrication of monodisperse microcapsules.

The production of PNIPAM microcapsules using NIPAM monomer was confirmed by employing Fourier transform infrared spectroscopy (FT-IR), which is a powerful tool to identify specific chemical bonds of the surface. Both NIPAM monomer and PNIPAM were analyzed, and the characteristic absorbance bands are marked with numbers and arrows as shown in Figure 3(B). Three distinctive absorbance peaks from NIPAM were observed at 917, 962, and 985 cm^−1^, and these peaks indicate the vibration of the C=C double bond. Moreover, the broad peak around 2,970 and 3,295 cm^−1^ indicates asymmetric −CH_2_ stretching and secondary amide N-H stretching. After polymerization of NIPAM and converted into PNIPAM, the chemical structure were clearly observed at 1,388 cm^−1^ (deformation of methyl group), 1,459 cm^−1^ (-CH_3_ and −CH_2_ deformation), 1,542 cm^−1^ (secondary amide N–H stretching), 1,631 cm^−1^ (secondary C=O stretching), 2,854 cm^−1^ (−CH_3_ symmetric stretching), 2,929 cm^−1^ (asymmetric −CH_2_ stretching), 2,975 cm^−1^ (asymmetric –CH_3_ stretching), and 3,286 cm^−1^ (secondary amide N–H stretching and bending) [7,22,23]. Comparing the NPIAM and PNIPAM, the three distinct C=C peaks from NIPAM disappeared and could not be observed in the PNIPAM FT-IR spectrum. Moreover, other major peaks, except for the C=C peaks, were still observed due to the similar chemical structures between NIPAM and PNIPAM. This result indicated the successful polymerization of NIPAM and fabrication of PNIPAM-based microcapsules using the microfluidic device.

To demonstrate the biological feasibility of PNIPAM microcapsules, EGFP-displayed *BT* spores were employed for further investigation. In addition, the *BT* spores were mixed with monomer and injected to produce microdroplets and the microorganism was encapsulated inside of the microcapsules after polymerization. The black small dots, which are encapsulated *BT* spores inside the highly transparent microdroplets, were easily observed using an optical microscope as shown in Figure 3.

**Figure 4.**
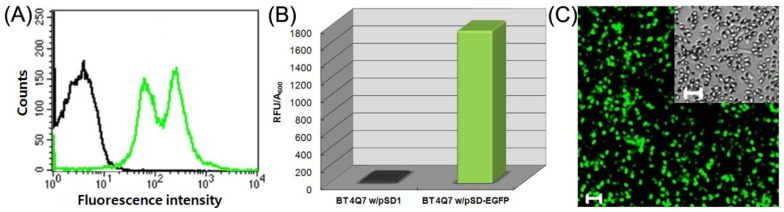
Spore-display of EGFP. (**A**) Flow cytometry analysis. Black line, *BT* spores harboring pSD1 as a negative control; green line, *BT* spores harboring pSD-EGFP; (**B**) Fluorescence assay. 1, *BT* spores harboring pSD1 as a negative control; 2, *BT* spores harboring pSD-EGFP; (**C**) Confocal microscopy analysis. The inset shows an optical microscopic image. Scale bars represent 5 μm.

As a first step towards developing our bioactive microcapsules, we sought to engineer the InhA-mediated spore-surface display of EGFP as a model protein. The EGFP-displayed *BT* spores were confirmed by flow cytometry (Figure 4(A)), fluorescence assay (Figure 4(B)) and confocal microscopy (Figure 4(C)). Specific fluorescence signals of the spores were observed in EGFP-displayed *BT* spores, indicating that green fluorescence was observed only with EGFP-displayed *BT* spores.

**Figure 5.**
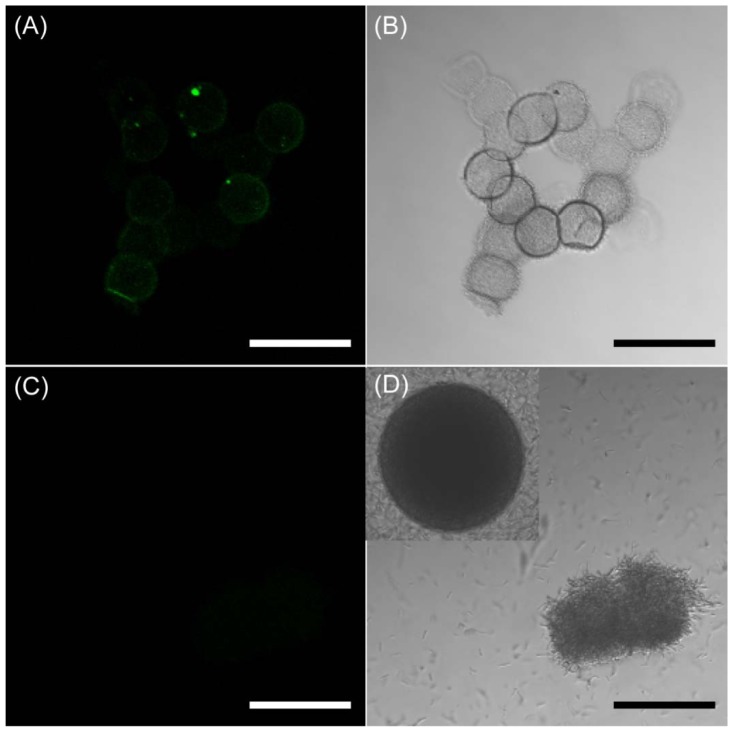
*BT* spore encapsulated in the hydrogel microcapsules. Fluorescent image of *BT* spore inside of microcapsules before the germination (**A**) and its optical image (**B**); Fluorescent image of microcapsules after the germination (**C**) and its optical image (**D**). Scale bars represent 100 μm.

For the confirmation of the encapsulation of EGFP-displayed *BT* spores in the microcapsules *BT* spore-encapsulated PNIPAM microcapsules were collected and investigated the fluorescent characteristics by confocal microscopy. As shown in Figure 5(A), the strong green fluorescent signal which is derived from encapsulated *BT* spores was observed from the fluorescent image. The highly transparent and monodisperse microcapsules were also obtained as shown in Figure 5(B). To investigate the viability of the encapsulated spores and subsequently shuttle into vegetative cells, we transfer the *BT* spores-encapsulated microcapsules were placed in Luria-Bertani (LB) medium for germination and incubated at 37 °C for 24 h. Once germinated, the vegetative cells did not display EGFP on their surface anymore. Nonetheless, it is noteworthy that the fluorescence of vegetative cells became weaker under the germination condition. In particular, the fluorescent and optical property change would be the strong evidence that the *BT* spores were converted into vegetative cells by the germination in microdroplets. In these reasons, we investigated the fluorescent and optical changes. The *BT* spores maintained their viability under the microencapsulating conditions and were successfully germinated into vegetative cells. In addition, there were no fluorescent signals in the microcapsules as shown in Figure 5(C). The spatially included microstructures of vegetative cells (live cells) were observed after 24 h of incubation, and some free vegetative cells were observed (Figure 5(D)). In addition, the highly transparent microcapsules were also converted into dark-gray color microcapsules. The darkness of the inside of microcapsule indicates that vegetative cells are agglomerated in the microcapsules. Some microcapsules were covered with vegetative cells as shown in Figure 5(D). Once the vegetative cells were growing and packing inside of the microcapsules, the hydrogels were flexible enough to break out the cells from the microcapsules. These results suggest that the strategy present herein should be useful in generating microstructure of any microbial cells by spatially addressing their spores within microdroplets.

## 4. Conclusions

We developed a novel method for producing bioactive microcapsules which encapsulated a biological species, *BT* spores, in a one-step process using a microdroplet-based microfluidic system. The size of microdroplets is mainly dependent on the both CP and DP flow rate applied to the microfluidic device. For the protection of *BT* spores from the surrounding environment, PNIPAM was used as a bio-inert material to produce the microcapsules. The viability of encapsulated *BT* spores was confirmed by the culture of the produced droplets for shuttling into vegetative cells after the spore germination. This method could provide an accurate, efficient and robust means to prepare bioactive microdroplets for droplet-based drug screening, biosensors, and biotransformations. Furthermore, it can be potentially applicable to develop whole-cell biosensors, having the potential to be developed into a rapid, high-throughput, field-portable method for the detection of biological and environmental samples.

## Supplementary Material
