# Supplementary material for: Synthesis of Bioactive Microcapsules Using a Microfluidic Device
Source: Sensors (Basel). 2012 Jul 26;12(8):10136–47. doi: 10.3390/s120810136 (PMC3472820; doi:10.3390/s120810136)
Supplement: Supplementary file 1 [file sensors-12-10136-s001.pdf]

OPEN ACCESS

sensors

ISSN 1424-8220

www.mdpi.com/journal/sensors

Supplementary Information

Synthesis of Bioactive Microcapsules Using a Microfluidic Device. *Sensors* 2012, *12*, 10136–10147

Byeong Il Kim [1,2,†], Soon Woo Jeong [1,2,†], Kyoung G. Lee [1], Tae Jung Park [3], Jung Youn Park [4], Jae Jun Song [5], Seok Jae Lee [1,*] and Chang-Soo Lee [2,*]

[1] Center for Nanobio Integration & Convergence Engineering (NICE), National Nanofab Center, 291 Daehak-ro, Yuseong-gu, Daejeon 305-806, Korea; E-Mails: kbiset@nnfc.re.kr (B.I.K.); swjeong@nnfc.re.kr (S.W.J.); kglee@nnfc.re.kr (K.G.L.)

[2] Department of Chemical Engineering, Chungnam National University, 220 Gung-Dong, Yuseong-gu, Daejeon 305-764, Korea

[3] Department of Chemistry, Chung-Ang University, 84 Heukseok-ro, Dongjak-gu, Seoul 156-756, Korea; E-Mail: tjpark@cau.ac.kr

[4] Biotechnology Research Division, National Fisheries Research & Development Institute (NFRDI), 408-1 Sirang-ri, Gijang, Busan 619-705, Korea; E-Mail: jypark@nfrdi.go.kr

[5] Microbe-based Fusion Technology Research Center, KRIBB, 1404 Sinjeong-dong, Jeongeup, Jeonbuk 580-185, Korea; E-Mail: jjsong@kribb.re.kr

[†] These authors contributed equally to this work.

* Authors to whom correspondence should be addressed; E-Mails: sjlee@nnfc.re.kr (S.J.L.); rhadum@cnu.ac.kr (C.-S.L.); Tel.: +82-42-879-9722 (S.J.L.); Fax: +82-42-879-9609 (S.J.L.); Tel.: +82-42-821-5896 (C.-S.L.); +82-42-822-8995 (C.-S.L.).

Received: 8 June 2012; in revised form: 4 July 2012 / Accepted: 18 July 2012 / Published: 26 July 2012

Figure S1. (**A**) Schematic illustration of microfluidic device and its dimensions; (**B**) Photograph of microfluidic device.

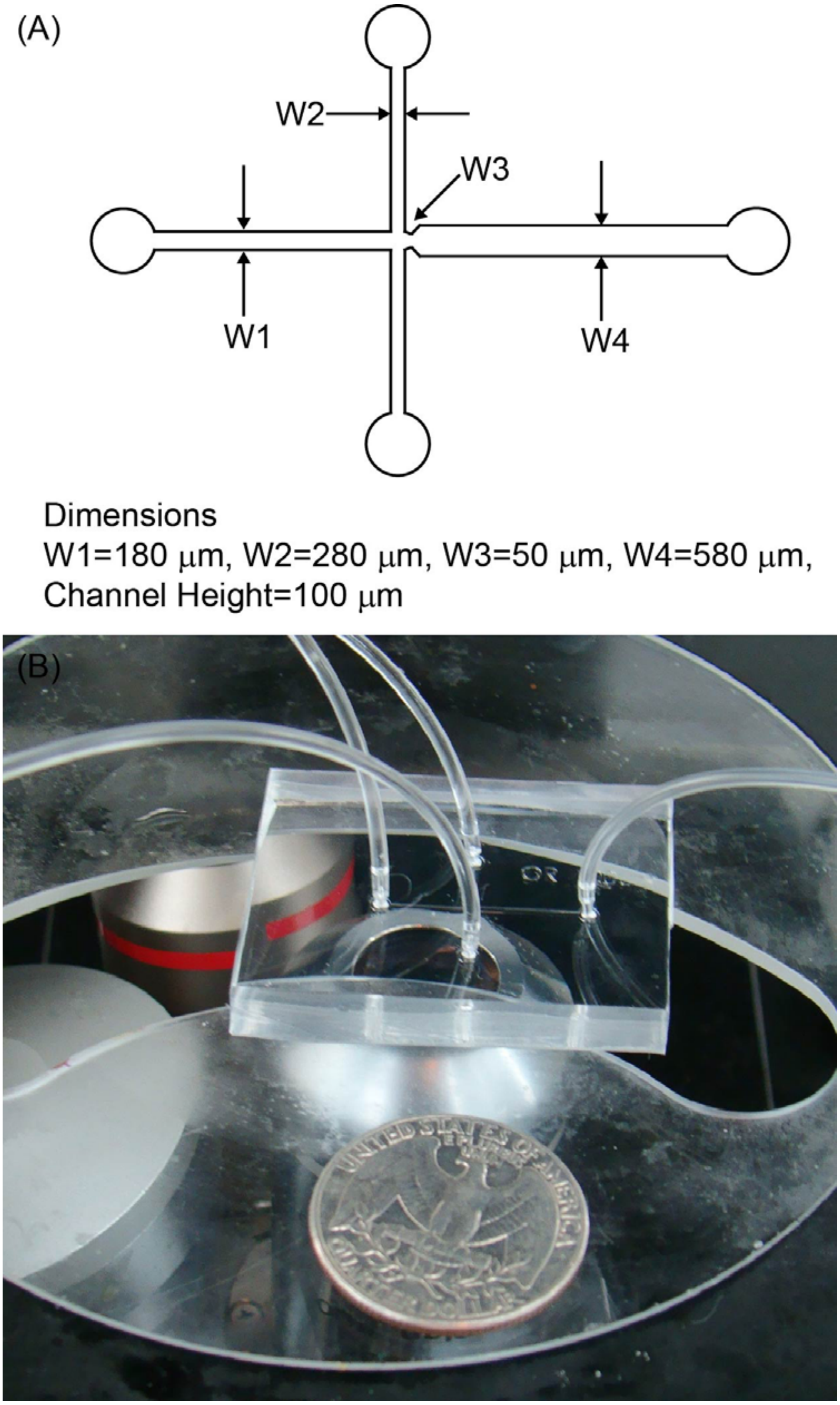

Figure S2. Photomicrographs showing the droplets of different sizes at the fixed DP (1 μL/min) with five different flow rates of CP. (**A**) Q_{CP1} = 1 μL/min; (**B**) Q_{CP2} = 2 μL/min; (**C**) Q_{CP3} = 3 μL/min; (**D**) Q_{CP4} = 4 μL/min; (E) Q_{CP5} = 5 μL/min. All scale bars are 500 μm.

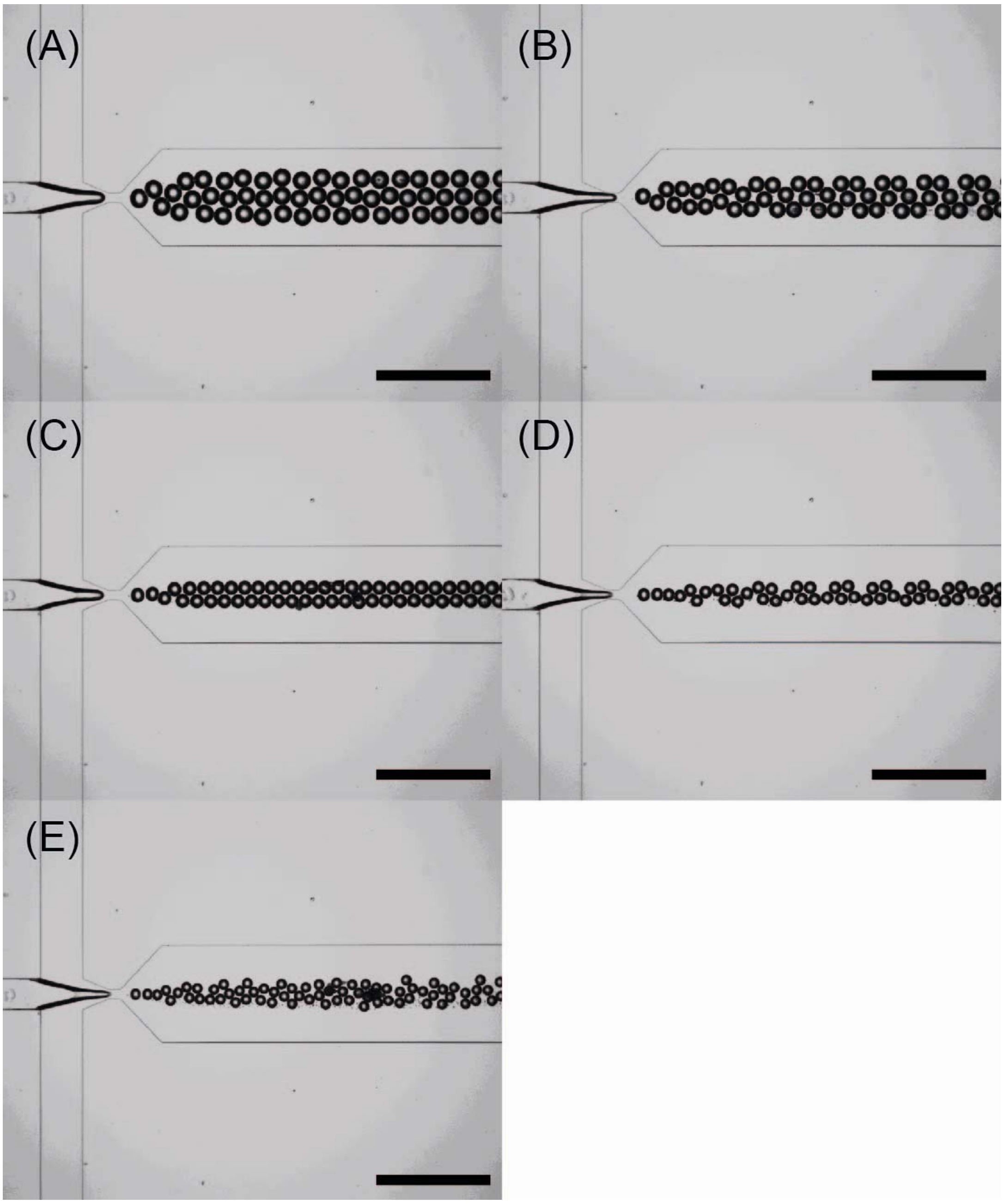

Figure S3. Photomicrographs showing the droplets of different sizes at the fixed DP (2 μL/min) with five different flow rates of CP. (**A**) Q_{CP1} = 1 μL/min; (**B**) Q_{CP2} = 2 μL/min; (**C**) Q_{CP3} = 3 μL/min; (**D**) Q_{CP4} = 4 μL/min; (**E**) Q_{CP5} = 5 μL/min. All scale bars are 500 μm.

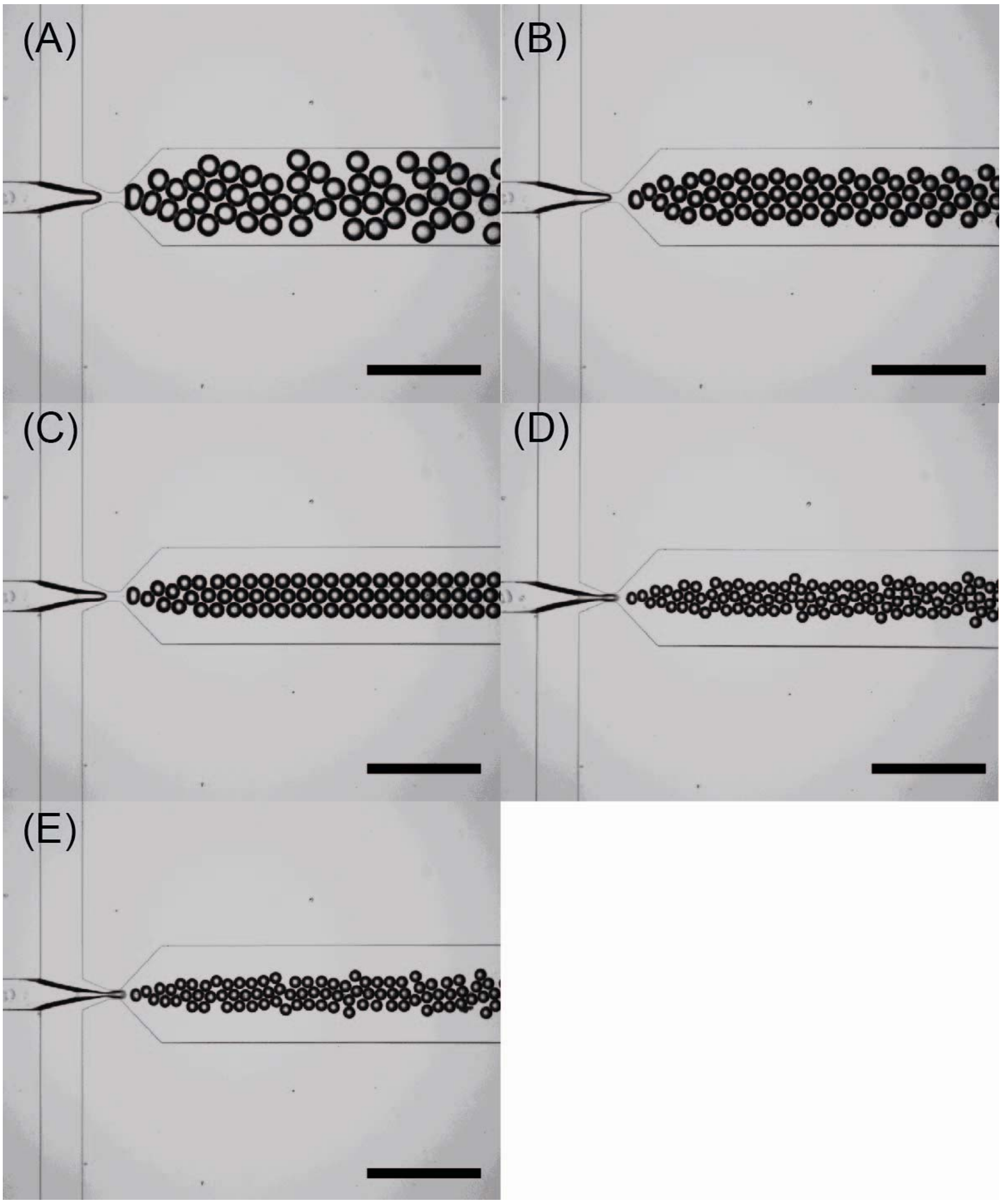

Figure S4. Photomicrographs showing the droplets of different sizes at the fixed DP (3 µL/min) with five different flow rates of CP. (**A**) Q_{CP1} = 1 µL/min; (**B**) Q_{CP2} = 2 µL/min; (**C**) Q_{CP3} = 3 µL/min; (**D**) Q_{CP4} = 4 µL/min; (**E**) Q_{CP5} = 5 µL/min. All scale bars are 500 µm.

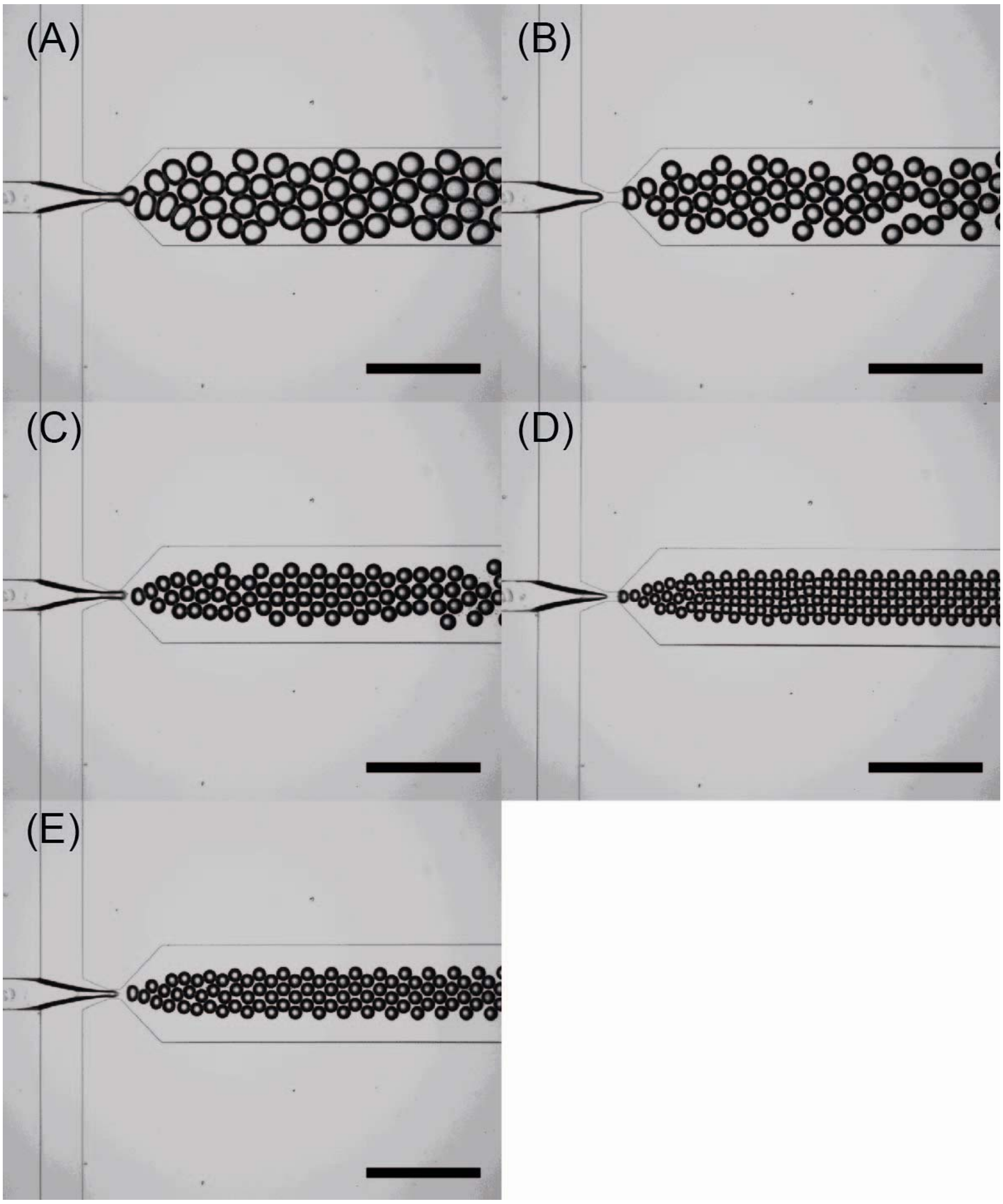

Figure S5. Photomicrographs showing the droplets of different sizes at the fixed DP (4 µL/min) with five different flow rates of CP. (**A**) Q_{CP1} = 1 µL/min; (**B**) Q_{CP2} = 2 µL/min; (**C**) Q_{CP3} = 3 µL/min; (**D**) Q_{CP4} = 4 µL/min; (**E**) Q_{CP5} = 5 µL/min. All scale bars are 500 µm.

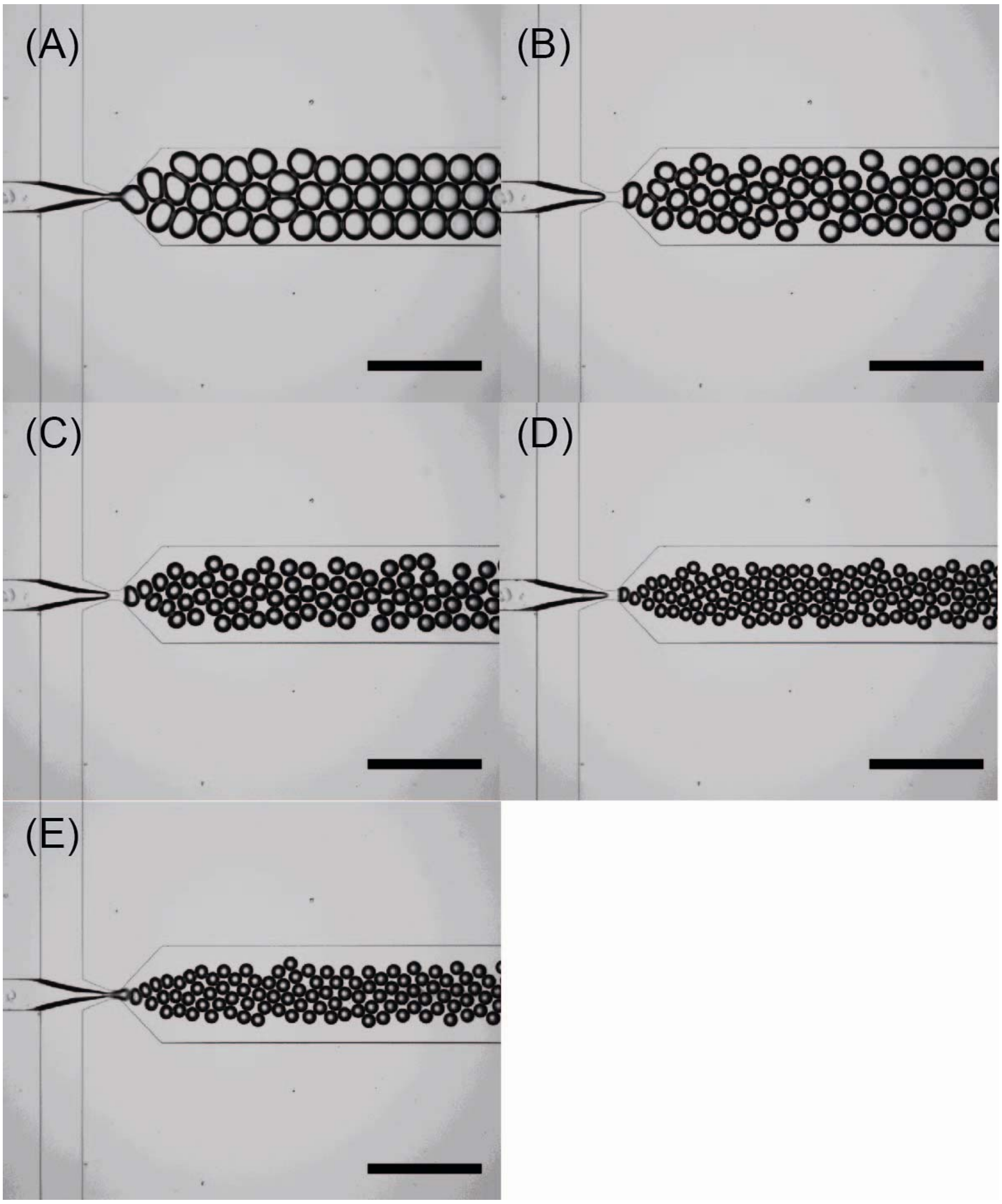

© 2012 by the authors; licensee MDPI, Basel, Switzerland. This article is an open access article distributed under the terms and conditions of the Creative Commons Attribution license (http://creativecommons.org/licenses/by/3.0/).
